# On the significance of the different geometrical and dosimetric parameters in microbeam and minibeam radiation therapy a retrospective evaluation

**DOI:** 10.3389/fonc.2024.1449293

**Published:** 2024-09-27

**Authors:** Josie May McGarrigle, Kenneth Richard Long, Yolanda Prezado

**Affiliations:** ^1^ Department of Physics, Imperial College London, London, United Kingdom; ^2^ Science and Technology Facilities Council (STFC), Rutherford Appleton Laboratory, Oxford, United Kingdom; ^3^ Institut Curie, Université Paris-Saclay, Centre national de la recherche scientifique (CNRS) UMR3347, Inserm U1021, Signalisation radiobiologie et cancer, Orsay, France; ^4^ Université Paris-Saclay, Centre national de la recherche scientifique (CNRS) UMR3347, Inserm U1021, Signalisation radiobiologie et cancer, Orsay, France; ^5^ New Approaches in Radiotherapy Lab, Center for Research in Molecular Medicine and Chronic Diseases (CIMUS), Instituto de Investigación Sanitaria de Santiago de Compostela (IDIS), University of Santiago de Compostela, A Coruña, Spain; ^6^ Oportunius Program, Galician Agency of Innovation (GAIN), A Coruña, Spain

**Keywords:** SFRT, MBRT, MRT, normal tissue sparing., spatially fractionated radiation therapy, Minibeam, Microbeam, biological response

## Abstract

**Introduction:**

Spatially Fractionated Radiation Therapy (SFRT) is an unconventional therapeutic approach with the potential to disrupt the classical paradigms of conventional radiation therapy. The high spatial dose modulation in SFRT is believed to activate distinct radiobiological mechanisms which lead to remarkable increases in normal tissue tolerance. To make optimal use of SFRT and its benefits, a deeper understanding of the biological response and its relationship with the complex dosimetric and geometric components of SFRT is essential.

**Method:**

A retrospective evaluation of preclinical studies was conducted to gain insight into the dosimetric and geometric parameters that are most correlated with normal tissue response. Current literature evaluates the response of tissue to MBRT and MRT according to various end points, e.g. the level of desquamation, degree of necrosis, or the amount of malcalcification. A set of metrics was developed to allow a quantitative comparison of these results.

**Results:**

The strongest correlations were observed with the doses in both the peaks and valleys as well as the ratio of the area covered by the peak over the total area. This emphasises the geometry of the beam. MBRT challenged previous uniform dose-distribution paradigms by highlighting the critical role of Peak Dose alongside Valley Dose in tissue sparing whereas MRT underscores the significant influence of geometric beam parameters on tissue preservation.

**Discussion:**

The data exhibits variability in the results obtained using different animal models and endpoints and additional research is warranted to explore the trends observed in this study under controlled conditions.

## 1 Introduction

Radiation therapy is one of the most efficient methods of cancer treatment. Treatments conventionally use broad beams and aim to deliver a homogeneous dose distribution at the target. Despite the remarkable advances in dose conformality, normal tissue tolerance continues to compromise the efficiency of treatment in a number of cases, such as bulky radioresistant tumours. To overcome these difficulties, various radiotherapy techniques are being explored. One promising technique is Spatially Fractionated Radiation Therapy (SFRT) in which alternating regions of high and low doses are used.

A significant reduction in normal-tissue toxicity has been reported in the SFRT treatment of both patients and small animal experiments (1–4). There are several forms of SFRT: GRID therapy (5), Lattice therapy (LRT) (6), Microbeam Radiation Therapy (MRT) (7) and Minibeam Radiation Therapy (MBRT) (8). A significant number of patients have been treated with GRID and LRT (3, 4). MRT and MBRT are still in the preclinical stage.

Most of the preclinical data was acquired with MRT and MBRT because of the convenient size and spacing of the narrow (submillimeter) beams used during rodent irradiations, although a few experiments using GRID and LRT were also carried out in tumour bearing animals (9, 10). Toxicity studies were only performed in MRT and MBRT. For this reason, the study presented here focuses on exploring MRT and MBRT, both of which have been shown to cause less toxicity to normal tissue than conventional broad beam radiotherapy (4).

MRT uses spatially fractionated beams in which each microbeam has a micrometer or submicrometer (≤ 100*µ*m, typically 25-100*µ*m) width (4). The use of thicker microbeam widths were observed to yield dose distributions that were not influenced by cardiac pulsations (4). This discovery prompted the study of MBRT, using widths ranging between 500 and 700*µ*m (4).

Current literature predicts that both MRT and MBRT are capable of treating a tumour effectively while minimising adverse toxicities (11–13). In a recent retrospective evaluation of preclinical studies in MRT and MBRT, Valley Dose was shown to be the parameter most correlated with increased lifespan (14) post-SFRT, but to date, there has been no investigation into which dosimetric or geometric parameter has the most significant influence over normal-tissue sparing. This review study presents an overview of data collected from a selection of experiments that investigated how the toxicities observed post-MRT and post-MBRT depend on the spatial configuration or dosimetry of the beam.

## 2 Materials and methods

This review study was constructed to analyse the normal-tissue response of MRT and MBRT in small animal models to determine which geometric and dosimetric parameters have the most effect.

### 2.1 Search criteria

**Figure 1:**
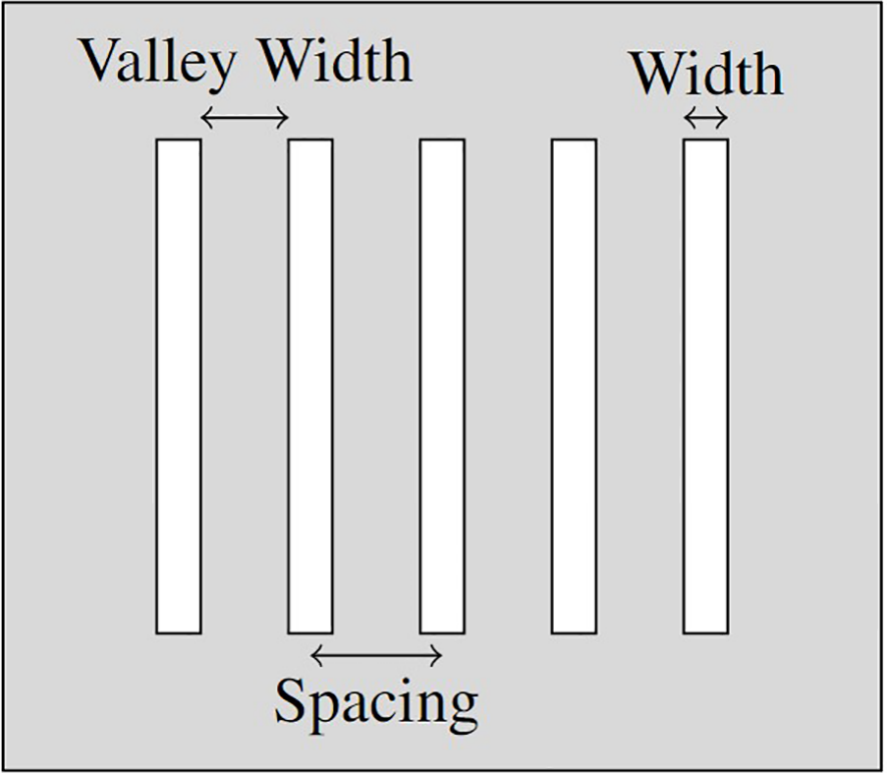
Mechanical collimator diagram annotated with geometric properties.

The parameters identified as potentially correlated with the sparing of healthy tissue in SFRT treatment can be divided into two categories; Geometric Parameters (see, **Figure 1**) and Dosimetric Parameters, as follows:

• The Geometric Parameters (see **Figure 1**):

 – Width (µm)

  _*_ the width of each beam segment (the collimator gap width);

 – Spacing (µm)

  _*_ the centre-to-centre (c-t-c) spacing between adjacent beam segments;

 – Valley Width (µm)

  _*_ the edge-to-edge spacing between adjacent beam segments;

 – % Peak Dose

  _*_ the percentage of width compared to c-t-c spacing (indicates the % of volume covered by the peak dose):

% Peak Dose = $\frac{\text{Width}}{\text{Spacing}}\times 100$ ;

• The Dosimetric Parameters:

 – Volume Average Dose (Gy)

  _*_ the average dose across the tissue volume;

 – Peak Dose (Gy)

  _*_ the dose received in the peaks of the dose distribution;

 – Valley Dose (Gy)

  _*_ the dose received in the valleys of the dose distribution;

 – PVDR (Peak-Valley-Dose-Ratio)

  _*_ the peak to valley dose ratio: $\text{PVDR}=\frac{\text{Peak}\;\text{Dose}}{\text{Valley}\;\text{Dose}}$ .

### 2.2 Selection criteria

Data from papers published before May 2024 were considered for inclusion in the present study. 20 papers were included, 11 of which used MRT as the therapy modality (11, 15–24) and 9 of which used MBRT (12, 25–32). The following criteria were used to select the data to be included in the study:

- Only a single fraction of unidirectional MRT or MBRT was used in the study;
- The study reports Average Dose, Peak Dose, Valley Dose, PVDR, Width, Spacing or Valley Width;
- The biological response of normal-tissue to MRT and MBRT is recorded in the study;
- Peak dose used in the study is ≤ 700Gy (previous studies into MRT record long-term alterations as Peak Dose exceeds 700Gy (33), thus these results were labelled ‘toxic’ and excluded to reduce the dataset to therapeutically relevant results only);
- The experiment in each study was carried out *in-vivo* using small animal models, **Table 1** lists the specific models used; and
- MRT or MBRT was exclusively used in each experiment.

**Table 1:** A table of tissue types/tumour types in selected experiments.

Modality	Tumour	Species	Irradiation Area	Reference
MBRT	None	Mouse	Leg	(25)
MBRT	None	Rat	Brain	(26–30)
MBRT	None	Mouse	Brain	(31)
MBRT	RG2 Rat Glioma cells	Rat	Brain	(12, 32)
MRT	None	Mouse	Leg	(15)
MRT	None	Mouse	Back	(16)
MRT	None	Mouse	Brain	(17)
MRT	None	Rat	Brain	(11, 18–22)
MRT	None	Mouse	Spine	(23)
MRT	EMT6 Murine Mammary Carcinoma	Mouse	Leg	(24)

The study evaluated each paper using PICO (population, intervention, comparison, outcome) as a search strategy tool. The categories used to collect data for this systematic review study are listed in **Table 2**. A more extensive description of each study and the corresponding PICO details are listed in the **Supplementary Materials**. After the data was curated, a search was made for correlations between the reported endpoints and the geometric/dosimetric parameters.

**Table 2:** Population, Intervention, Comparison, Outcome (PICO) search strategy used to select relevant experiments.

Population	Intervention
Small animal *in-VIVO* models	Single unidirectional SFRT irradiation (dose ≤ 700*Gy*)
Comparison	Outcome
Control group/pre-radiation	Biological response described/quantified

**Figure 2:**
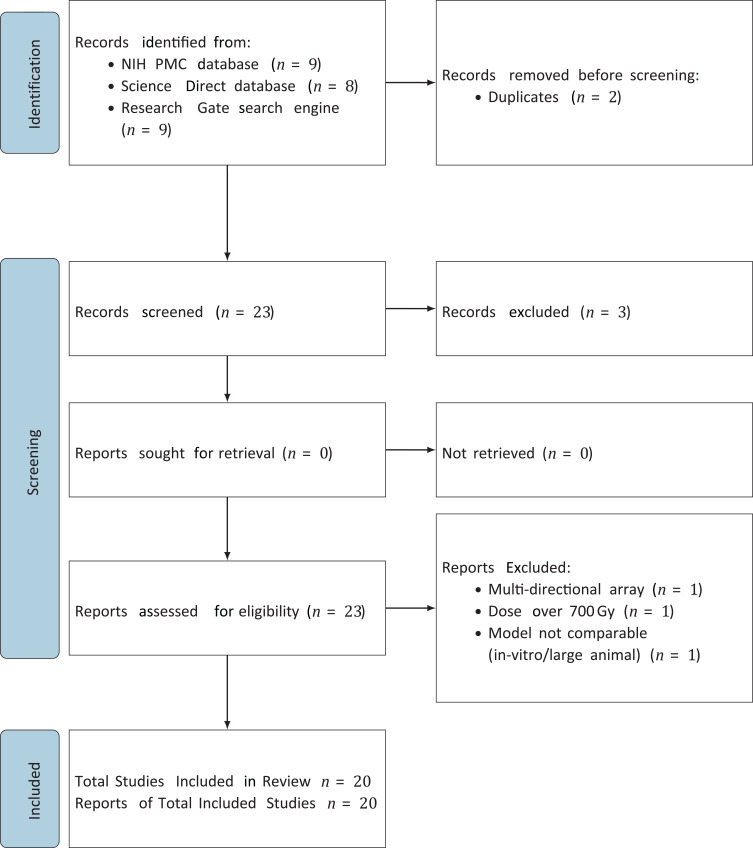
PRISMA flow diagram.

PRISMA (Preferred Reporting Items for Systematic reviews and Meta-Analyses) guidelines were used in this systematic review. **Figure 2** presents a PRISMA flow diagram, which outlines the study selection process. This diagram was created to provide a clear and detailed account of how studies were identified, screened, and included in the review.

### 2.3 Data analysis

A set of metrics was developed for this review study to allow a quantitative comparison of the varying endpoints. Scores were assigned to reflect the degree to which normal-tissue sparing had been observed. Scores were allocated based on the criteria defined in **Table 3**.

**Table 3:** Scoring system used to quantify the experimental biological responses, labelled ‘Normal Tissue Sparing Score’ also known as NTSS.

Score	Sparing	Biological Response
1	None	No normal-tissue sparing, high damage e.g. permanent deformation/death
2	Low level	Little normal-tissue sparing, moderate damage e.g. moist desquamation/paralysis/cognitive impairment
3	Moderate	Some normal-tissue sparing observed, noticeable damage e.g. moderate necrosis/patchy moist desquamation
4	Fair	Normal-tissue sparing observed, minimal/reparable damage e.g. mild dry desquamation/depilation
5	Great	Complete normal-tissue preservation, no damage e.g. complete cognitive function/skin unaffected

The end-point in each experiment was evaluated, converting the qualitative result into a number between 1 and 5 (mid category scores were also awarded if a result fell between two categories). The bivariate Pearson’s correlation coefficient, *r*, was calculated to quantify the degree of any correlation between the chosen parameter and the amount of normal-tissue sparing. Pearson’s correlation coefficient is given by:


(1) $$r=\frac{\sum_{i=1}^{n}(x_{i}-\overset{¯}{x})\left(y_{i}-\overset{¯}{y}\right)}{\sqrt{\sum_{i=1}^{n}{\left(x_{i}-\overset{¯}{x}\right)}^{2}}\sqrt{\sum_{i=1}^{n}{\left(y_{i}-\overset{¯}{y}\right)}^{2}}};$$

where *n* is the number of measurements in the sample, the $x_{i}$ are the values of the geometric/dosimetric parameters, the $y_{i}$ are the associated quantitative outcomes, and the mean of the $x_{i}$ and $y_{i}$ are $\overset{¯}{x}$ and $\overset{¯}{y}$ respectively. |r|>0.5 is categorised as a “strong correlation”, *r* in the range 0.3<|r|≤0.5 is categorised as a moderate correlation, and |r|≤0.3 is categorised as a weak correlation.

A confidence-level analysis to establish the degree to which the null hypothesis, that the outcome is uncorrelated with the geometric/dosimetric parameter, can be rejected was carried out by calculating the test statistic, *t*, given by:


(2) $$t=r{\left(\frac{n-2}{1-r^{2}}\right)}^{\frac{1}{2}}\;.$$

where r is the Pearson correlation coefficient and *n* is the size of the sample. For the null hypothesis, *r* = 0 and *t* follows the Student’s *t* distribution with n−2 degrees of freedom. The confidence level, or “*p*-value”, was evaluated as the probability that a value with magnitude ≤|t| would occur by chance. For this review study, statistical significance is characterised as a *p*-value of less than 0.05.

As a cross-check, the standard deviation of the data points from the value expected based on the null hypothesis $\left(\sigma_{null}\right)$ , that the points and geometric/dosimetric parameter are uncorrelated and the alternative hypothesis (σ) , that the points are correlated with the geometric/dosimetric parameter, were calculated. The standard deviations are defined by:


(3) $$\sigma_{null}=\sqrt{\frac{\sum \;{\left(y_{i}-\overset{¯}{y}\right)}^{2}}{n-1}}\;;$$

(4) $$\sigma =\sqrt{\frac{\sum \;{\left(y_{i}-y_{\text{est}}\right)}^{2}}{n-2}}\;;$$

where $y_{\text{est}}$ is the estimate of the end-point score obtained from the line of best fit, and *n* is the number of data points. If $\sigma <\sigma_{null}$ , it is more likely that the end point is correlated with the geometric/dosimetric parameter, while, if $\sigma_{null}<\sigma$ , it is more likely that the data and geometric/dosimetric parameter are uncorrelated.

To examine the trends observed in the data, each scored endpoint was evaluated and plotted as a function of the most significant geometric/dosimetric parameter of the dataset. A straight line fit was performed on each graph to allow the correlation to be visualised. The straight-line fit was used to determine the 95% confidence interval CI and the 95% prediction interval PI by evaluating:


(5) $$\text{CI}=\hat{y}\pm t_{\text{crit}}\cdot \sigma \sqrt{\frac{1}{n}+\frac{{\left(x-\overset{¯}{x}\right)}^{2}}{{\left(\hat{x}-\overset{¯}{x}\right)}^{2}}}\;;\text{and}$$

(6) $$\text{PI}=\hat{y}\pm t_{\text{crit}}\cdot \sigma \sqrt{1+\frac{1}{n}+\frac{{\left(x-\bar{x}\right)}^{2}}{{\left(\hat{x}-\bar{x}\right)}^{2}}};$$

where $\hat{y}$ is the NTSS, $t_{\text{crit}}$ is the statistic of interval confidence also known as the critical value of the *t* distribution (34), *σ* is the squared deviation of the end point values, x is an array of evenly spaced x values for the range of each geometric/dosimetric parameter, $\overset{¯}{x}$ is the mean of the geometric/dosimetric parameters.

## 3 Results

SFRT tissue-sparing results were divided into MBRT (Section 3.1) and MRT (Section 3.2). Previous studies concluded that survival analysis post MBRT and MRT produced differing trends for the set of dosimetric and geometric parameters defined in this study (2). As a result, each modality has been investigated separately.

### 3.1 Minibeam (MBRT) results

**Figure 3:**
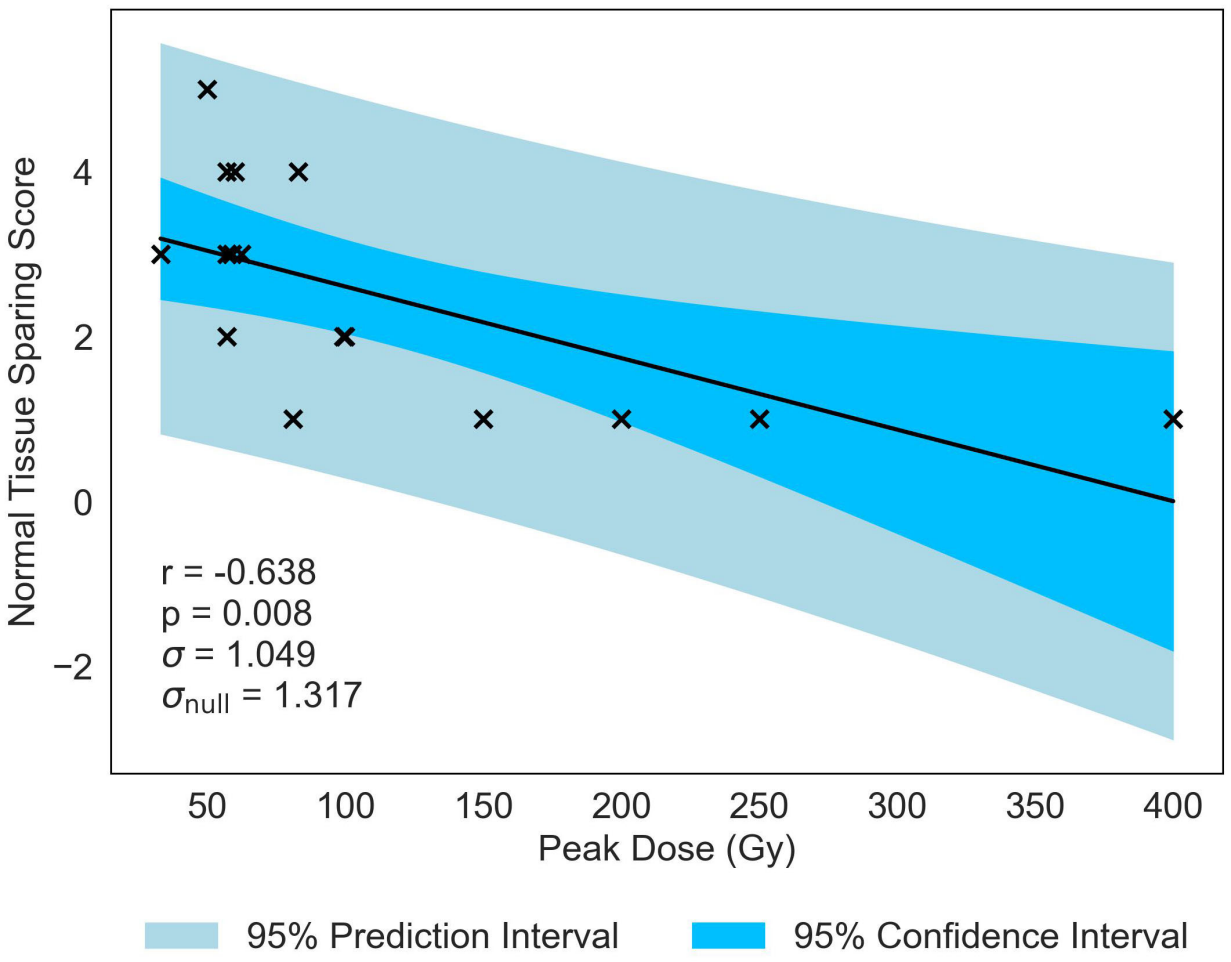
Normal-tissue Sparing score plotted against the most significant and strongest geometric/dosimetric parameter for MBRT, Peak Dose. There is a strong negative correlation between the parameters, illustrating that an increase in the dose at the peak regions will increase the damage to the normal-tissue.

Normal-tissue sparing post MBRT was found to be most strongly correlated with Peak Dose (see **Table 4**). With the strong negative correlation of *r* = −0.638 and statistically significant *p*-value of *p* = 0.008, the data indicates that, as Peak Dose increases, there is more damage to the normal tissue. **Figure 3** illustrates the negative correlation, presenting a line of best fit accompanied by confidence and prediction intervals.

**Table 4:** Statistical analysis for normal-tissue sparing post MBRT.

Parameter	*r*	*p*	*σ*	$\sigma_{\text{null}}$
Volume Average Dose (Gy)	-0.620	0.010*	1.070	1.317
Peak Dose (Gy)	-0.638	0.008*	1.049	1.317
Valley Dose (Gy)	-0.573	0.020*	1.117	1.317
% Peak Dose	-0.395	0.145	1.238	1.298
PVDR	0.123	0.649	1.352	1.317
Width (um)	0.124	0.649	1.352	1.317
Spacing (um)	0.326	0.235	1.274	1.298
Valley Width (um)	0.353	0.196	1.260	1.298

Key: r - Pearson Correlation Coefficient, *p* - *p*-value (significance), σ - residual standard deviation, $\sigma_{\text{null}}$ - residual standard deviation for a 0 correlation. Statistically significant correlations are identifiable by an asterisk. All values are rounded to 3 decimal places.

**Figure 4:**
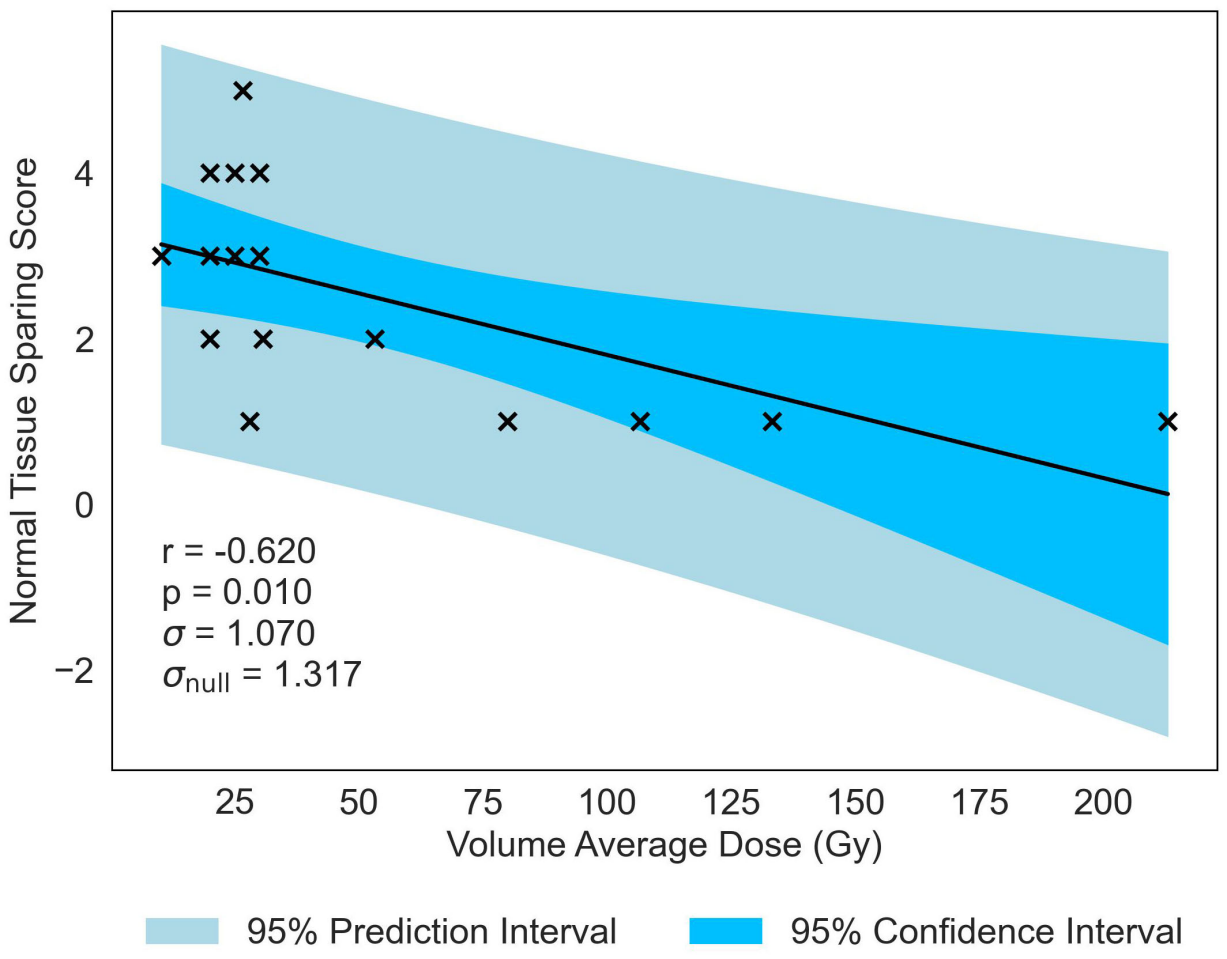
Normal-tissue Sparing Score plotted against the second most significant/strongest geometric/dosimetric parameter for MBRT, Volume Average Dose. There is a negative correlation between the parameters, illustrating that an increase in average dose administered across the tissue volume will increase the damage to the normal-tissue, replicating the trend seen in **Figure 3** with Peak Dose.

Similar to previous literature (2), Valley Dose is established as a critical parameter, significantly correlated with NTSS, demonstrating a similar negative trend (*r* = −0.573, *p* = 0.020). This observed trend naturally extends to Volume Average Dose, given the interdependence of the three parameters, resulting in a statistically significant correlation with NTSS (*r* = −0.620, *p* = −0.010), displayed in **Figure 4**. Volume Average Dose emerges as the second most critical parameter for MBRT, even surpassing the significance of Valley Dose. A multivariate analysis was carried out to confirm this interdependence (see **Supplementary Materials**). The analysis suggests a correlation between increasing dose and damage to normal tissue. The negative correlation is presented in **Figure 4**. $\sigma_{\text{null}}>\sigma$ for all statistically significant results, indicating that the correlation gives a reasonable description of the data.

### 3.2 Microbeam (MRT) results

**Figure 5:**
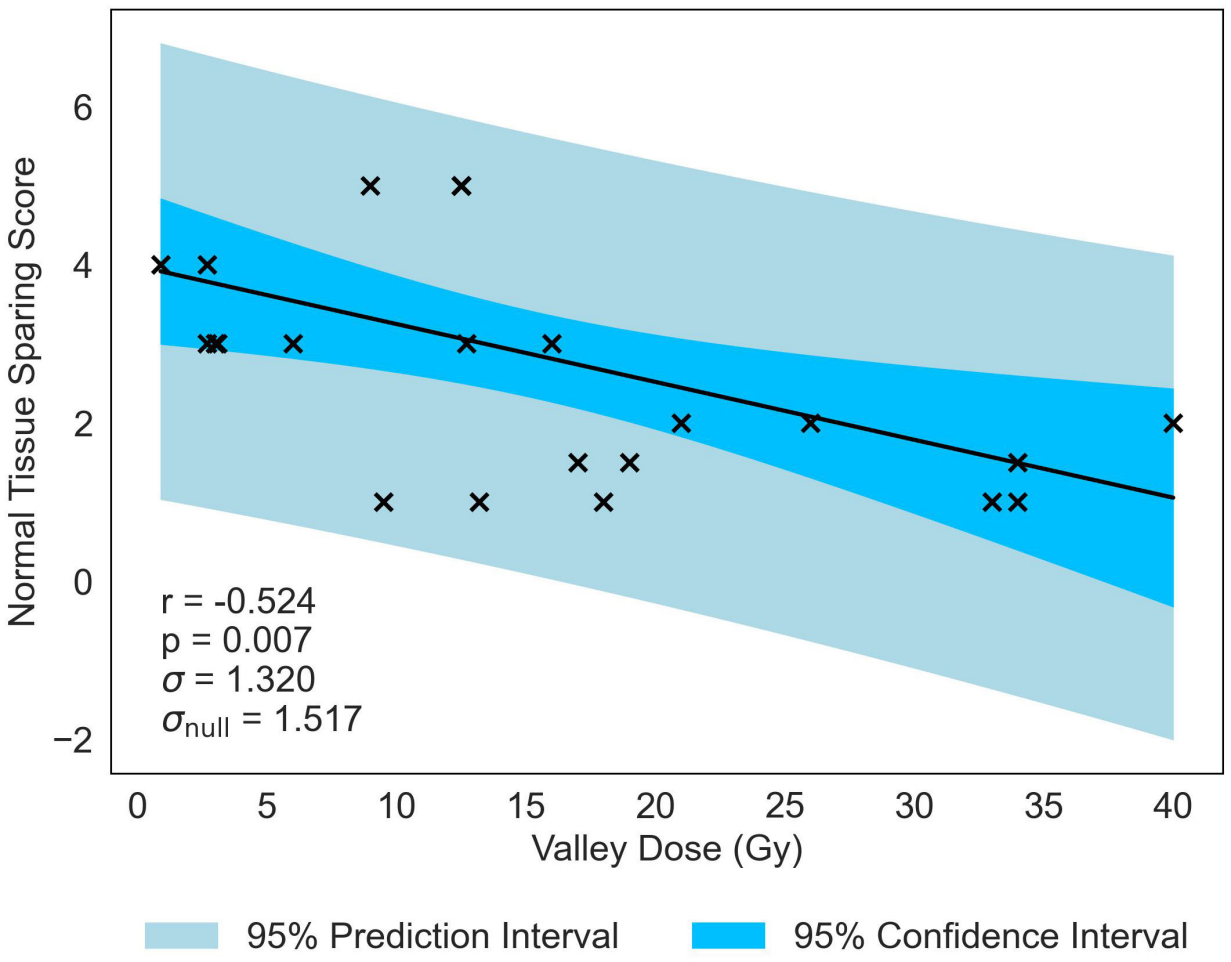
Normal-tissue Sparing Score plotted against the most significant/strongest geometric/dosimetric parameter for MRT, Valley Dose. There is a negative correlation between the parameters, illustrating that a decrease in the dose to the valley regions will increase the damage to the normal-tissue and suggests a reduction in sparing.

Normal-tissue sparing post MRT is most significantly correlated to Valley Dose (*r* = −0.524, *p* = 0.007), see **Table 5**. The pronounced negative trend associated with Valley Dose, as displayed in **Figure 5**, drives the hypothesis that this trend contributed to the statistically significant positive correlation observed with PVDR (*r* = 0.405, *p* = 0.044), though the multivariate analysis shows only a moderate correlation (see **Supplementary Materials**).

**Table 5:** Statistical analysis for normal-tissue sparing post MRT.

Parameter	*r*	*p*	*σ*	$\sigma_{\text{null}}$
Volume Average Dose (Gy)	-0.177	0.397	1.525	1.517
Peak Dose (Gy)	0.064	0.709	1.639	1.619
Valley Dose (Gy)	-0.524	0.007*	1.320	1.517
% Peak Dose	-0.409	0.013*	1.499	1.619
PVDR	0.405	0.044*	1.417	1.517
Width (um)	0.058	0.736	1.640	1.619
Spacing (um)	0.367	0.027*	1.528	1.619
Valley Width (um)	0.399	0.016*	1.506	1.619

Key: r - Pearson Correlation Coefficient, *p* - *p*-value (significance), σ - residual standard deviation, $\sigma_{\text{null}}$ - residual standard deviation for a 0 correlation. Statistically significant correlations are identifiable by an asterisk. All values are rounded to 3 decimal places.

**Figure 6:**
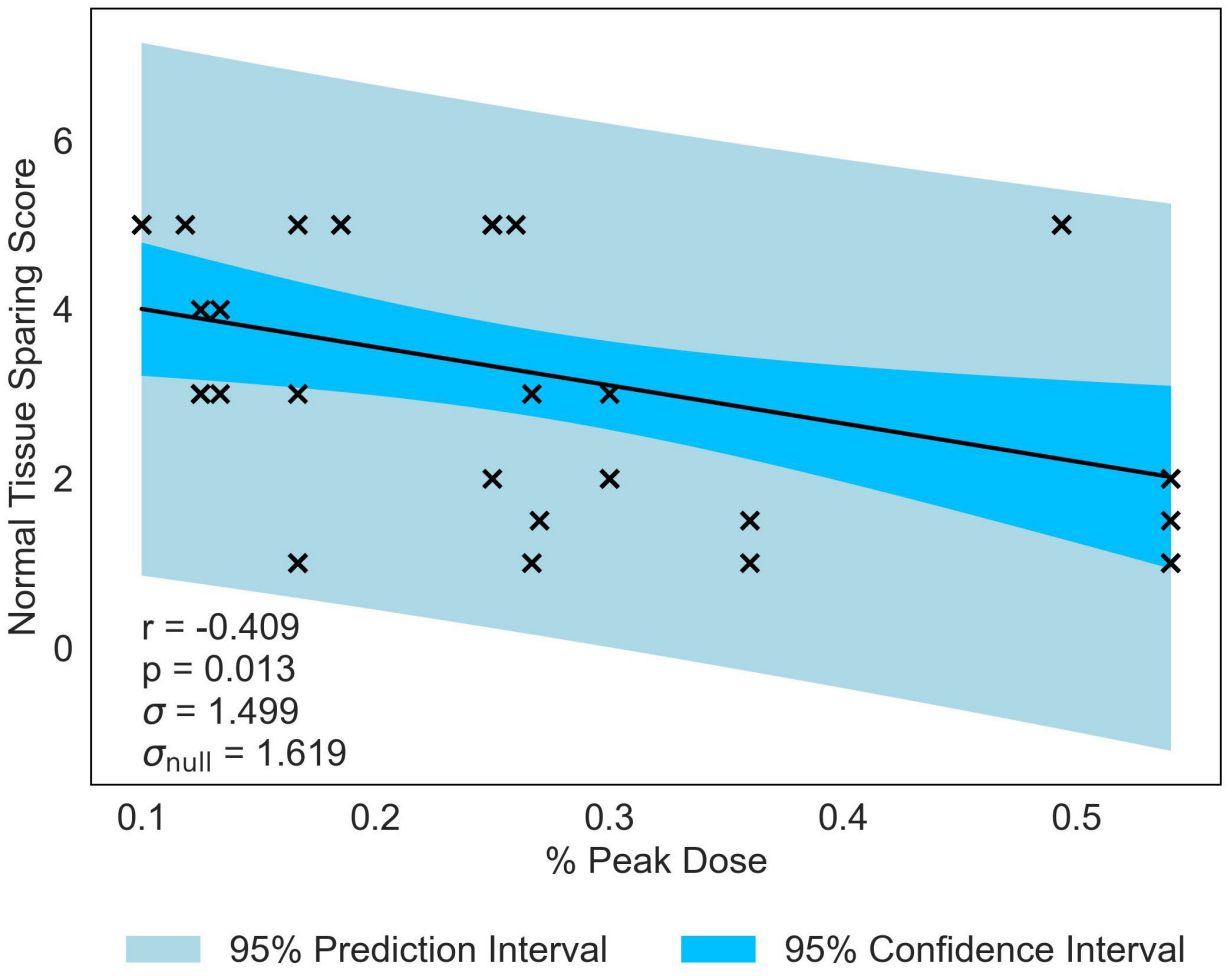
Normal-tissue Sparing Score plotted against the joint most significant/strongest geometric/dosimetric parameter for MRT, % Peak Dose. There is a negative correlation between the parameters, illustrating that a decrease in % Peak Dose will increase the damage to the normal-tissue and suggests a reduction in sparing.

The second most significant NTSS correlation is with % Peak Dose, producing a statistically significant *p*-value of *p* = 0.013 and moderate correlation of *r* = −0.409 (see **Figure 6**). This data suggests that, as the area occupied by the Peak Dose is reduced, there is an increasing preservation of normal-tissue. Valley Width and Spacing also have statistically significant correlations (*r* = 0.399, *p* = 0.016 and *r* = 0.367, *p* = 0.027 respectively), indicating that, in contrast to MBRT, tissue-sparing is likely to be driven by geometric configuration in MRT.

For all statistically significant correlations, the standard deviation for the null hypothesis, $\sigma_{\text{null}}$ , is greater than the standard deviation, σ , evaluated with the fitted correlation. This indicates that the correlation gives a reasonable description of the data.

## 4 Discussion

**Figure 7:**
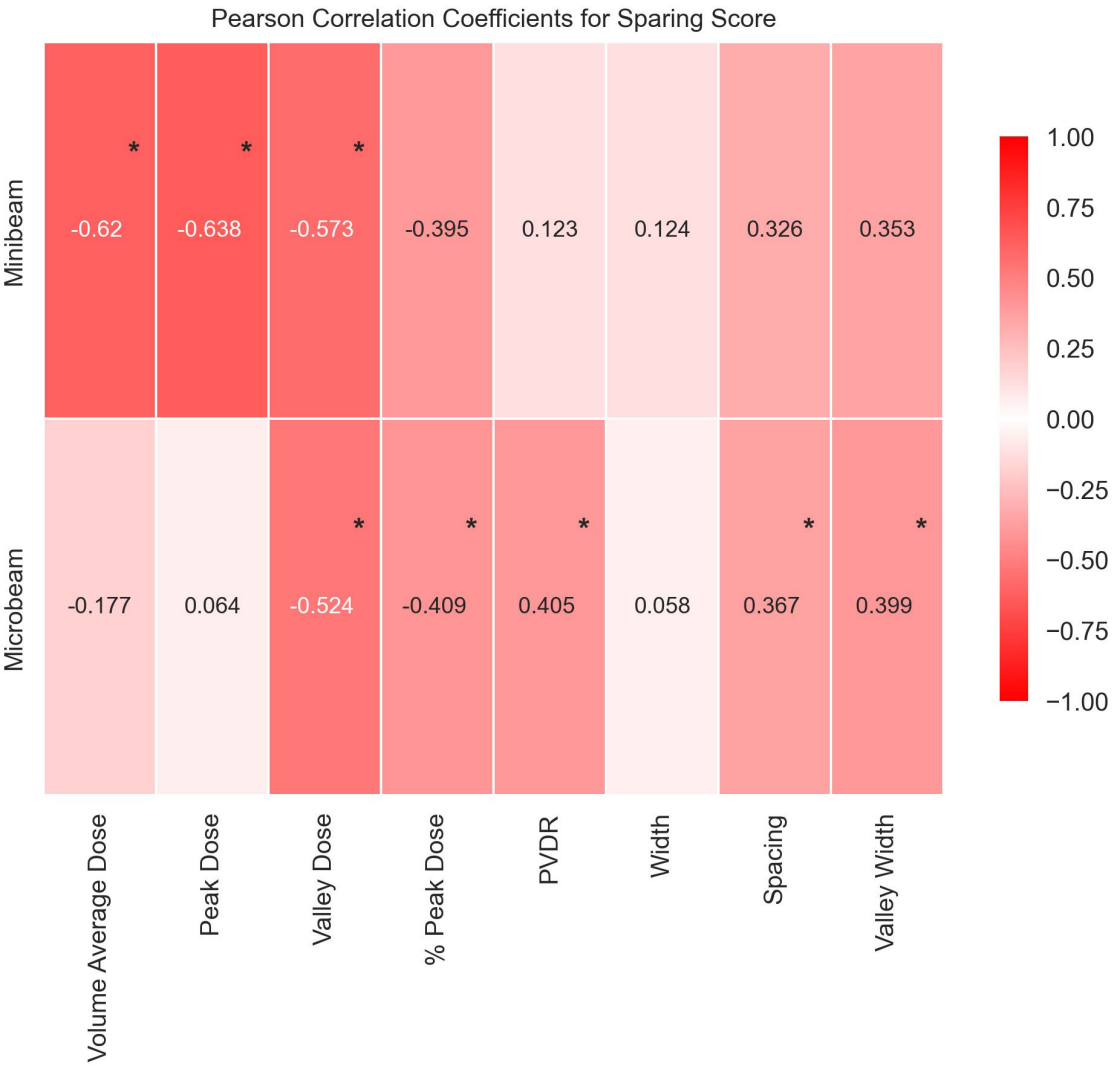
Graph to display Pearson’s Correlation Coefficients in heat map form to show the correlations between each geometric/dosimetric parameter and the sparing score for MBRT and MRT. The values range between -1 and 1, where the extremities (closest to -1 and 1) have the deepest colour and the weakest correlations (closer to 0) have a weak colour. Statistically significant correlations are identifiable by an asterisk.

In this comprehensive analysis of tissue sparing post Microbeam Radiation Therapy (MRT) and Minibeam Radiation Therapy (MBRT), significant insights were gained into the role of geometric and dosimetric beam parameters. An overview of the study is presented in **Figure 7**. Summary data tables are found in the **Supplementary Materials** along with full descriptions of the end points and scores.

The MBRT analysis challenges previous assumptions that only Valley Dose is important for tissue sparing by identifying Peak Dose as a critical parameter, see **Figure 7**. All dosimetric parameters have statistically significant negative correlations with tissue sparing after MBRT treatment, underscoring the crucial role of dose parameters in influencing tissue response to MBRT. Specifically, the significant negative correlations suggest that higher doses, particularly in peak and volume-average distributions, result in increased damage to normal tissues. This observation highlights the need for precise dose modulation to balance therapeutic efficacy with normal tissue preservation in MBRT treatment regimes.

Unlike MBRT, the MRT analysis underscores the importance of geometry, particularly emphasising the sensitivity of normal tissues to the proportion of the irradiation volume covered by the Peak Dose. The existing body of literature has predominantly emphasised the significance of dosimetric parameters over geometric factors as critical determinants in radiation therapy outcomes. This study identifies the pivotal role geometric beam parameters have in optimising MRT treatment protocols and mitigating normal tissue toxicity.

The study also presents statistically significant negative correlations between Valley Dose and tissue sparing in both MRT and MBRT treatment regimes. The existing literature underscores the significance of Valley Dose as a crucial factor for increased lifespan in small animal models (14). These findings suggest a link between tissue sparing and lifespan increase, hinting at broader physiological implications beyond immediate treatment outcomes.

Observing the data globally, a pattern emerges regarding the influence of beam width on tissue response. If the beam width is sufficiently narrow, the peak dose has a minimal impact on normal tissue response, with the critical parameter being the dose received in valley regions and the area covered by the valleys. As the beam width broadens, the dose received by the peaks starts to play a more significant role in sparing normal-tissue, surpassing Valley Dose as the critical parameter. These observations are crucial for the application and optimisation of advanced radiotherapy techniques such as MBRT, GRID, and Lattice therapy.

This retrospective review highlights key parameters that may influence normal tissue sparing in SFRT. The main limitations of the study include variations in dose rates and in the time points at which biological responses to SFRT are recorded. MRT treatment regimes are carried out at extremely high dose rates (35). In the current body of literature, it is impossible to disentangle a FLASH effect (36) from tissue sparing due to MRT in isolation. However, given the extremely high doses used in MRT, the presence of a FLASH effect seems unlikely. Even if a FLASH effect were contributing to normal tissue sparing, it would have minimal impact on the variation in biological responses due to changes in dosimetric and geometric parameters, as most experiments were conducted under consistent beam conditions at the same facility. Moreover, most MBRT experiments were performed at conventional dose rates, with a few early experiments using higher dose rates at synchrotron facilities. The results seem largely unaffected by the absence of ultra-high dose rates.

The timing of endpoint evaluations can be influential, as the severity of radiation-induced damage can vary significantly over time. Unfortunately, not all studies included in this review provide sufficient information on the time points used for assessing biological responses, making it difficult to conduct a statistically meaningful analysis of how the timing of response measurements might influence the reported outcomes. To address this, future studies should adopt standardised time points for endpoint evaluation, or at the very least, ensure that time points are clearly reported to facilitate meaningful comparisons across studies.

## 5 Conclusion

Spatially Fractionated Radiation Therapy (SFRT) is a technique that divides the beam into evenly spaced segments, designed to spare normal-tissue. This review study analyses the normal-tissue response of SFRT in small animal models in order to investigate the effect of two modalities, MRT and MBRT. The current body of literature indicates that both Microbeam Radiotherapy (MRT) and Minibeam Radiotherapy (MBRT) promise effective treatment while mitigating cellular toxicities, with MBRT demonstrating a potentially enhanced sparing effect. This review study systematically identified the critical geometric/dosimetric parameters of MRT and MBRT for sparing healthy tissue. MRT emphasises the impact of Valley Dose and the geometric properties of the beam, while MBRT challenges previous literature by highlighting the critical role of the Peak Dose parameter for tissue sparing in MBRT treatment. In summary, this review study highlights key geometric/dosimetric factors that impact the normal-tissue sparing properties of MRT and MBRT, discovering an emphasis on Peak Dose, Valley Dose and geometry. The global data on MRT and MBRT indicate that for narrow beam widths, Valley Dose predominantly influences normal tissue response. As beam width increases, Peak Dose becomes more critical to the sparing of normal tissue. These insights can aid in improving treatment planning for better radiotherapeutic results. Future research with larger, balanced datasets (homogeneous in animal models, set-up and end-points) is needed to gain a more detailed understanding of how dosimetry and geometry influences treatment outcomes in these innovative approaches.

## Data Availability

The original contributions presented in the study are included in the article/**Supplementary Material**. Further inquiries can be directed to the corresponding author.
